# Biomechanics of Periventricular Injury

**DOI:** 10.1089/neu.2019.6634

**Published:** 2020-04-09

**Authors:** Zhou Zhou, Xiaogai Li, Svein Kleiven

**Affiliations:** Division of Neuronic Engineering, Royal Institute of Technology (KTH), Huddinge, Sweden.

**Keywords:** brain–ventricle interface, finite element analysis, fluid-structure interaction, periventricular injury

## Abstract

Periventricular injury is frequently noted as one aspect of severe traumatic brain injury (TBI) and the presence of the ventricles has been hypothesized to be a primary pathogenesis associated with the prevalence of periventricular injury in patients with TBI. Although substantial endeavors have been made to elucidate the potential mechanism, a thorough explanation for this hypothesis appears lacking. In this study, a three-dimensional (3D) finite element (FE) model of the human head with an accurate representation of the cerebral ventricles is developed accounting for the fluid properties of the intraventricular cerebrospinal fluid (CSF) as well as its interaction with the brain. An additional model is developed by replacing the intraventricular CSF with a substitute with brain material. Both models are subjected to rotational accelerations with magnitudes suspected to induce severe diffuse axonal injury. The results reveal that the presence of the ventricles leads to increased strain in the periventricular region, providing a plausible explanation for the vulnerability of the periventricular region. In addition, the strain-exacerbation effect associated with the presence of the ventricles is also noted in the paraventricular region, although less pronounced than that in the periventricular region. The current study advances the understanding of the periventricular injury mechanism as well as the detrimental effects that the ventricles exert on the periventricular and paraventricular brain tissue.

## Introduction

Traumatic brain injury (TBI) is a substantial public health threat worldwide with an estimated more than 10 million patients with TBI each year.^1^ Pathological examinations of the patients with TBI^2–12^ and animal tests^13–24^ have frequently observed traumatic lesions in the periventricular region as one element of severe TBI. Periventricular injury is pathologically characterized as axonal injury along margins of the ventricles,^13,20^ subependymal hemorrhage at the ventricle wall,^5,6^ and intraventricular hemorrhage.^7^ It has been pioneeringly hypothesized by Holbourn^25^ that a strain concentration would occur near the ventricle wall during impact, which seemed explain the vulnerability of periventricular tissue. However, this hypothesis remains to be properly verified and the associated mechanical role that the ventricles play is yet to be better uncovered.

As surrogates of the human head, both physical models and finite element (FE) models are used to uncover the biomechanical influence of the ventricles on periventricular injury. However, consistent findings have not been reached to date. In addition to the seminal study by Holbourn^25^ that inferred that the ventricles might disturb the brain strain distribution, Ivarsson and co-workers^26^ quantitatively determined the influence of lateral ventricles by comparing the strain responses of two-dimensional (2D) physical models, one with a ventricle inclusion and one without. The strain responses were calculated by analyzing the trajectories of markers embedded within the brain substitute. It was found that the lateral ventricles had a strain-relieving effect on the tissues inferior and superior to the ventricles. Later, Ivarsson and co-workers^27^ further refined the physical model by incorporating an irregular skull base. More recently, several three-dimensional (3D) physical models were developed by Anata and colleagues^28^ to clarify the influence of ventricle shape on brain strain. It was concluded that the cerebral ventricles amplified strain near the corpus callosum and mitigated brainstem deformation.

Secondary to the prevalence of animal tests, several groups have developed FE models of animal heads to simulate the animal experiments that were known to cause periventricular axonal injury. Zhou and colleagues^29^ constructed 2D coronal models of porcine brain and simulated the animal test conducted by Ross and associates.^20^ It was concluded that the inclusion of ventricles was necessary to produce stress concentration in the periventricular region. Later, Al-Bsharat^30^ improved these 2D coronal models by introducing a sliding interface between the ventricles and brain. In parallel, Miller and co-workers^31^ coupled 2D axial models of porcine brain with animal tests to evaluate the capabilities of different modeling approaches in terms of replicating the topographic distribution of axonal injury and cortical contusion in the experiments. It was reported that, when modeling the ventricles as hollow cavities along with approximating the brain–skull interface as a sliding frictional interface, the predicted strain and von Mises stress patterns showed good correlation with experimental lesion distribution. Recently, a 3D rat model, developed by Antona-Makoshi,^32^ employed different modeling strategies to reproduce the rat experiments by Davidsson and Risling.^16^ Strain concentration was found around the ventricles, when the ventricles were left empty.

In addition to animal models, a handful of FE models of human head were also developed to illuminate the ventricles' influence on brain responses. By comparing the stress and strain predictions between two 3D models with different levels of anatomical differentiation, Zhou and colleagues^33^ found that the presence of the ventricles contributed to higher shear stress encompassing the ventricles. Contrarily, Nishimoto and Murakami^34^ reported that the ventricles and interpeduncular cistern had limited effects on the shear stress distribution in the brain, when studying the responses in the 2D coronal models of human head secondary to lateral impact.

Previous numerical studies suggest that a fluid representation of the cerebral ventricles along with appropriate treatment of the brain–ventricle interface is essential for accurate injury prediction, especially for periventricular injury.^31,33,34^ Among the aforementioned FE models, the brain–ventricle interface modeling approach varied from treating the ventricles as hollow cavities,^31,32^ merging the ventricles with the brain,^29,32–34^ to more a complicated contact algorithm defined between the ventricles and brain, such as tied-break contact^32^ and sliding contact.^30^ For the approach of treating the ventricles as hollow cavities, the incompressible nature of the intraventricular cerebrospinal fluid (CSF) is ignored. For the other approaches, the ventricles are consistently modeled as Lagrangian elements with different levels of shear modulus. These Lagrangian-based approaches were challenged by Zhou and co-workers,^35^ regarding their incapabilities of simulating the fluid properties of the CSF and potential fluid flow during impact.

In preference to the Lagrangian-based CSF representation, recent models have employed an arbitrary Lagrangian-Eulerian (ALE) multi-material formulation to describe the subarachnoid CSF combined with a fluid-structure coupling algorithm for the brain–skull interface.^36–39^ However, such a modeling strategy has not been implemented for the brain–ventricle interface to date, especially in traumatic scenarios. This may be partially attributed to a high-resolution description of the interfacial geometry being indispensable for fluid-structure interaction (FSI) implementation. Due to the limitation of mesh size, the ventricle boundary in the existing head models is either largely simplified,^40–43^ or jagged even with a mesh smoothing operation.^44–47^ Neither meets the requirement of an accurate description of the interfacial geometry amenable to FSI implementation.

Thus, the aim of the present work was to uncover the mechanism of periventricular injury. A 3D detailed FE model of human head with an accurate representation of cerebral ventricles was developed accounting for the fluid properties of the intraventricular CSF as well as its interaction with the brain. An additional model was developed by replacing the intraventricular CSF with a substitute with brain material. Both models were subjected to rotational accelerations with magnitudes suspected to induce severe diffuse axonal injury (DAI). By comparing the strain responses in both models, the biomechanical mechanism for the vulnerability of the periventricular tissue is better addressed.

## Methods

### Finite element head model

**FIG. 1.**
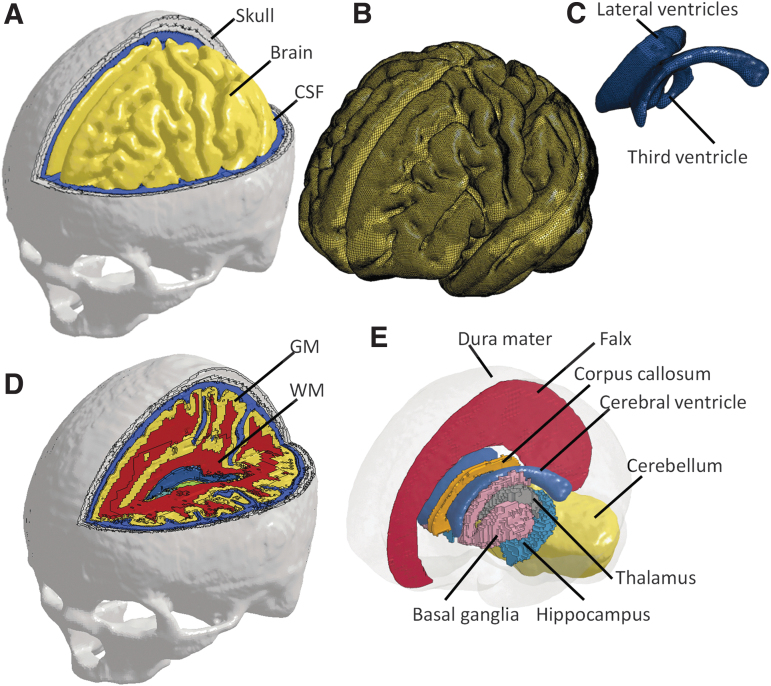
Isometric views of the FE model of human head. **(A)** Head model with the brain exposed. **(B)** Brain model. **(C)** Model of the cerebral ventricles. **(D)** Head model with the GM and WM visible. **(E)** Deep brain structures illustrated with the falx, cerebellum, and dura mater in translucency. For better illustration, FE meshes are only visible in the subfigures (b) and (c). CSF, cerebrospinal fluid; FE, finite element; GM, gray matter; WM, white matter.

The head model^48^ was developed from an averaged magnetic resonance imaging (MRI) head template, obtained from the database established by Fillmore and colleagues.^49^ The high-resolution T1- and T2-weighted images were segmented using the Freesurfer software.^50^ The segmentation was subsequently processed by the 3D SLICER software^51^ to obtain the surfaces of the skull, brain, and cerebral ventricles, all of which serve as input to the Hexotic software, generating all hexahedron elements based on an octree algorithm.^52^ The head model included the skull, subarachnoid CSF (i.e., CSF within the subarachnoid space), brain, and cerebral ventricles (i.e., lateral ventricles and third ventricle) (Fig. 1, upper row). This mesh generation process in combination with an appropriate mesh resolution allows preservation of morphological features of the brain (Fig. 1B) and cerebral ventricles (Fig. 1C). The volume ratio between the cerebral ventricles and brain was 1.9%, approximating the ratio range (1–4%) in adults.^53^ An in-house algorithm was developed to further classify the brain elements into different components based on the segmentation. The anatomically grouped brain included gray matter (GM), white matter (WM) (Fig. 1D), corpus callosum, cerebellum, thalamus, hippocampus, and basal ganglia (Fig. 1E). The falx and tentorium, which are invisible in the images, were manually created as shell elements based on the classification of brain tissue and anatomical illustrations. The pia mater was generated by finding the faces of brain elements. The resultant mesh of the model consisted of a total of 4.2 million hexahedral elements and 0.5 million quadrilateral elements. The parameters on the mesh quality are summarized in Appendix 1.

### Material properties

The material properties of each component are listed in Table 1, excluding the cerebral ventricles, which are detailed in the following section. The skull was modeled as rigid without separating the cortical bone from diploe bone.^47^ Although brain tissue was recognized as inhomogeneous and anisotropic, a thorough mechanical characterization was lacking. Thus, the brain was assumed to be a homogenous and isotropic structure with its material properties described by a second-order Ogden-based hyperelastic constitutive model.^43^ The subarachnoid CSF was modeled as an elastic fluid constitutive model and shared interfacial nodes with the brain and skull.^46^ Mechanical behaviors of the intracranial membrane were determined by the averaged material stress-strain curves from tissue experiments conducted by Aimedieu and Grebe^54^ for pia mater, and by Van Noort and co-workers^55^ for dura mater/falx/tentorium, respectively.

**Table 1.** Material Properties Used for the Head Model

Tissue	Young's modulus (MPa)	Density (kg/dm^3^)	Poisson's ratio
Skull^51^	6500	2.00	0.22
Brain^47^	Hyper-viscoelastic	1.04	∼0.5
Subarachnoid CSF^50^	K = 2.1 GPa	1.00	N/A
Dura mater/Falx/Tentorium^58^	Average stress-strain curve	1.13	N/A
Pia mater^59^	Average stress-strain curve	1.13	N/A

CSF, cerebrospinal fluid; K, bulk modulus; N/A, not applicable.

### Brain–ventricle interface modeling

Following the approach in previous publications,^26–28,33,34^ two models with and without an inclusion of the cerebral ventricles are presented (referred to as ventricle-model and no-ventricle-model, hereafter). Such a study design enables comparisons between the current study and previous publications.

**FIG. 2.**
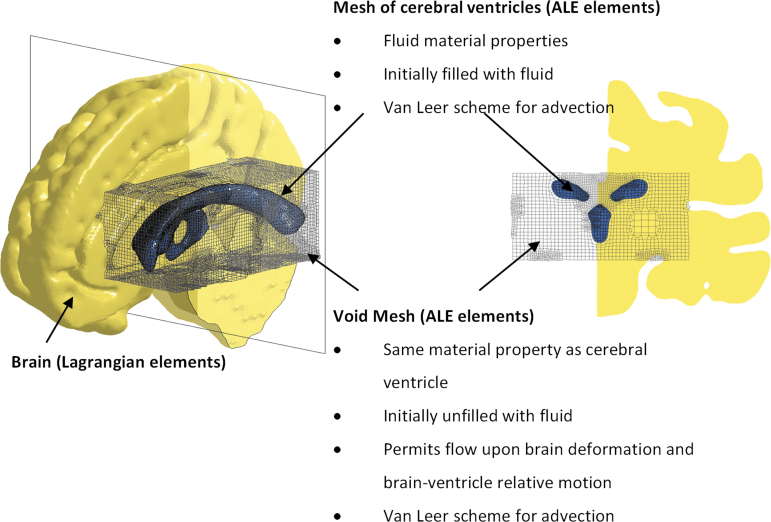
Mesh configuration and material properties of the brain–ventricle interface. Isometric view of the brain model with a cut-section plane (left) and the brain cut-section in coronal view (right). For better illustration, half of the brain is masked. The cerebral ventricles are shown as shaded elements and void mesh as wireframe elements. ALE, arbitrary Lagrangian-Eulerian.

For the ventricle-model, an FSI approach was used to simulate the brain–ventricle interface. To capture the fluid properties of the intraventricular CSF as well as the potential fluid flow during impact, the cerebral ventricles were modeled using an ALE multi-material formulation.^35^ Per the requirement of FSI implementation, any locations to which the fluid may potentially flow during the simulation had to be meshed. We expected that the intraventricular CSF could be transported to the space initially occupied by the deep brain structures due to brain deformation and brain–ventricle relative displacement. To account for that, additional meshes, referred to as void mesh in Figure 2, were generated in these regions, which initially overlapped with parts of the brain mesh. The void mesh was assigned the same material properties and element formulation as the cerebral ventricle elements, except for an extra void definition to ensure no fluid material was filled in the void mesh at its initial configuration. The motion of the ALE elements followed the mass-weighted averaged velocity in the ALE mesh.^35^

As elaborated by Zhou and co-workers,^35^ the ALE multi-material formulation advances the solution in time by dividing the operation into two steps, wherein the material is initially deformed in a Lagrangian step, followed by an advection step with a remapping of the element variables. In the Lagrangian step, the intraventricular CSF deformation was governed by the equation of state (EOS) for dilatational responses and constitutive equation for deviatoric responses with the associated formulations and material constants listed in Table 2. In the advection step, the element state variables were transported back to the reference domain with potential mass fluxes flowing within the mesh. Given its superiority in terms of numerical stability and advection accuracy, a second-order van Leer scheme was selected.^35^

**Table 2.** Material Constants for the Cerebral Ventricle

Equation of state	C (m/s)	S_1_	S_2_	S_3_	a	*γ*_0_
$P\text{ =}\frac{\rho_{0}C^{2}\mu \left[1\text{ + }\left(1-\frac{\gamma_{0}}{2}\right)\mu -\frac{a}{2}\mu^{2}\right]}{\left[1\;-\left(S_{1}-\;1\right)\mu -S_{2}\frac{\mu^{2}}{\mu \text{ + }1}\;-S_{3\;}\frac{\mu^{3}}{{\left(\mu \;-\;1\right)}^{2}}\right]};\mu \text{ = }\frac{V_{0}}{V}\;-1$	1482.9	2.1057	−0.1744	0.010085	0	1.2
Constitutive equation	γ (*Pa.s*)	*PC* (MPa)				
$\sigma_{ij}^{v}\text{ = }\gamma {\dot{\varepsilon }}_{ij}^{\prime }$	0.001	−22				

*P* is the pressure, *C*is the intercept of *v_s_-v_p_* curves with *v_s_* being the velocity of a shockwave traveling through the intermediary material and *v_p_* being the velocity of the shocked material; *S*_1_*, S*_2_, and *S*_3_ are the coefficients of the slope of the *v_s_-v_p_*curves, *γ*_0_ is the Gruneisen gamma, and *a* is the first order volume correction to *γ*_0_ *;V*_0_ is the initial volume; *V* is the instantaneous volume; $\sigma_{ij}^{v}$ is the deviatoric stress; *γ* is the dynamic viscosity; ${\dot{\varepsilon }}_{ij}^{\prime }$ is the deviatoric strain rate; *PC* is the cutoff pressure.

For the interface between the cerebral ventricles (ALE solid elements) and brain (Lagrangian solid elements), an FSI coupling algorithm was defined. The chosen algorithm allows sliding in the tangential direction as well as deliverance of tension and compression in the radial direction.^35^

For the no-ventricle-model, the ventricular cavities were filled by a substitute with brain material (Table 1). Given that the substitute was described by a Lagrangian formulation, the void mesh was not needed. Except for these, the material setting and undeformed mesh configuration of the no-ventricle-model were exactly the same as those of the ventricle-model.

Both models were validated against experimental data of brain–skull relative motion presented by Hardy and associates^56^ (see Appendix 2).

### Loading conditions

Two sinusoidal-shaped rotational impulses were imposed to both models in three different planes, respectively. One impulse peaked at 10 krad/s^2^ with a pulse duration of 5 msec (referred to as the 10 krad/s^2^-5 msec impulse, hereafter), whereas the other impulse peaked at 2500 rad/s^2^ with a pulse duration of 20 msec (referred to as the 2.5 krad/s^2^-20 msec impulse, hereafter). Both impulses approach the tolerance criterion for DAI in man proposed by Margulies and co-workers.^57^ Such loading choices were motivated by the histopathological observation that periventricular injury is frequently detected along with DAI.^6,58^ To ensure that the brain peak response was reached, which typically lags behind the peak acceleration impulse, the simulated durations were set to 30 msec for the simulations with rotational acceleration profiles of the 10 krad/s^2^-5 msec impulse, and 40 msec for the simulations with rotational acceleration profiles of the 2.5 krad/s^2^-20 msec impulse.

A constant stress integration scheme and hourglass control were used for all the head model components except for the cerebral ventricles. The hourglass energies were controlled to be lower than 10% of the total energy for each component in the models during the entire impact. The massively parallel processing version of LS-DYNA R11 was used with 256 processors requiring 52 h for the ventricle-model and 7 h for the no-ventricle-model for an impact of 30 msec.

### Statistical analysis

Following the approach of Miller and co-workers,^31^ first principal Green-Lagrangian strain was selected to evaluate brain injury. To avoid the potential numerical issue, the 95th percentile maximum principal Green-Lagrangian strain (referred to as MPS, hereafter) is presented, the same as the strategies in previous studies.^59,60^

**FIG. 3.**
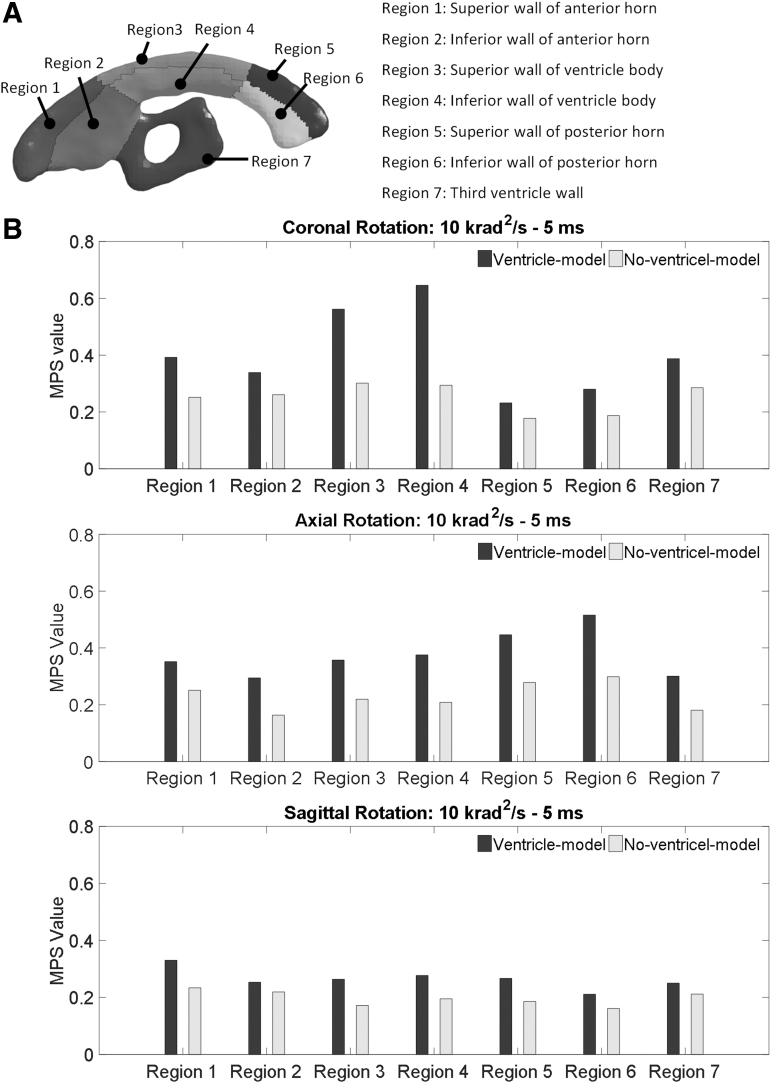
Comparison of the MPS values of the periventricular region in the ventricle-model and no-ventricle-model for the simulations with the rotational acceleration profiles being the 10 krad/s^2^-5 msec impulse. **(A)** Regional partition of the periventricular region. **(B)** The MPS values in the periventricular region for simulations of coronal rotation, axial rotation, and sagittal rotation, respectively. MPS, maximum principal Green-Lagrangian strain.

To statistically determine the ventricle influence on the strain responses, the MPS in the regions of interest (ROIs) in two models were analyzed with a Wilcoxon matched pairs signed rank test. The ROIs included seven subregions of the periventricular tissue (Fig. 3A) and four regions of the paraventricular tissue (Fig. 1E). The difference is considered significant for *p* < 0.05.

## Results

**FIG. 4.**
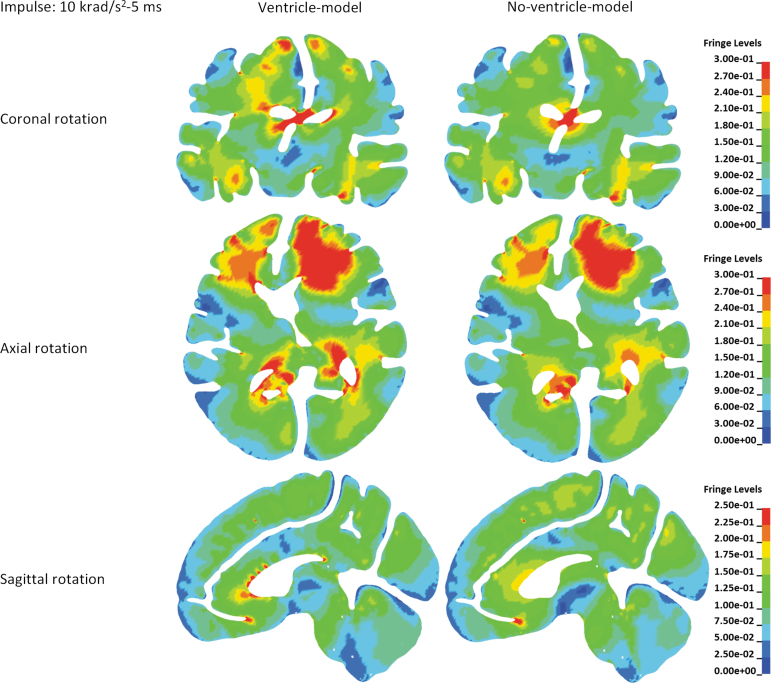
Comparison of the first principal Green-Lagrangian strain distribution between the ventricle-model and no-ventricle-model for the simulations with the rotational acceleration profiles being the 10 krad/s^2^-5 msec impulse. Fringe Levels represent first principal Green-Lagrangian strain. Upper row is the comparison of the first principal Green-Lagrangian strain distribution in coronal cross-sections for the simulations of coronal rotations. Middle row is the comparison of the first principal Green-Lagrangian strain distribution in axial cross-sections for the simulations of axial rotations. Lower row is the comparison of the first principal Green-Lagrangian strain distribution in sagittal cross-sections for the simulations of sagittal rotations.

**FIG. 5.**
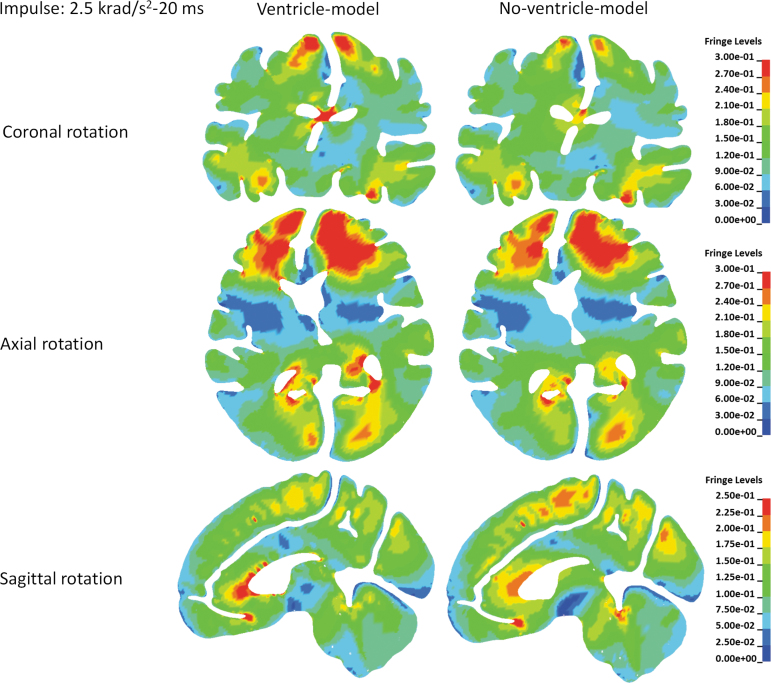
Comparison of the first principal Green-Lagrangian strain distribution between the ventricle-model and no-ventricle-model for the simulations with the rotational acceleration profiles being the 2.5 krad/s^2^-20 msec impulse. Fringe Levels represent first principal Green-Lagrangian strain. Upper row is the comparison of the first principal Green-Lagrangian strain distribution in coronal cross-sections for the simulations of coronal rotations. Middle row is the comparison of the first principal Green-Lagrangian strain distribution in axial cross-sections for the simulations of axial rotations. Lower row is the comparison of the first principal Green-Lagrangian strain distribution in sagittal cross-sections for the simulations of sagittal rotations.

Influence of the ventricle presence on the brain strain distribution is depicted in Figure 4 with the rotational acceleration profiles being the 10 krad/s^2^-5 msec impulse, and in Figure 5 with the rotational acceleration profiles being the 2.5 krad/s^2^-20 msec impulse. Similar strain distributions were noted in both models, except that distinct strain concentrations around the ventricles were exclusively predicted by the ventricle-model. Such concentrations were particularly evident in the interventricular region for the coronal rotations, anterior horn, and posterior horn for the axial rotations, and anterior horn for the sagittal rotations.

**FIG. 6.**
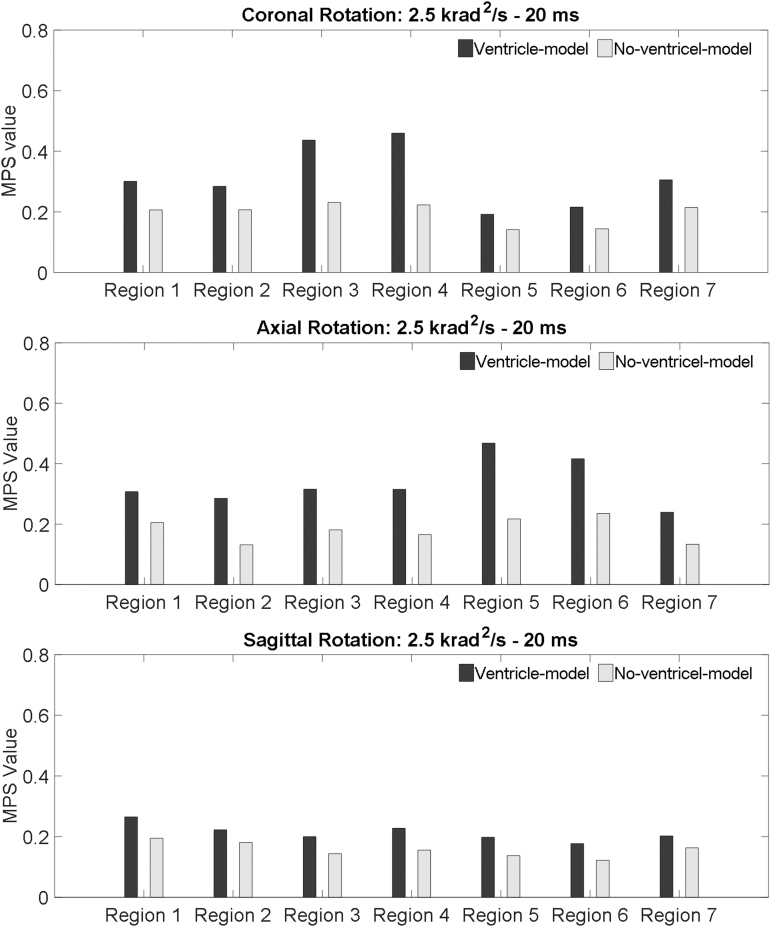
Comparison of the MPS values of the periventricular region in the ventricle-model and no-ventricle-model for the simulations of coronal rotation, axial rotation, and sagittal rotation, respectively. The rotational acceleration profiles are the 2.5 krad/s^2^-20 msec impulse for all the simulations. MPS, maximum principal Green-Lagrangian strain.

**FIG. 7.**
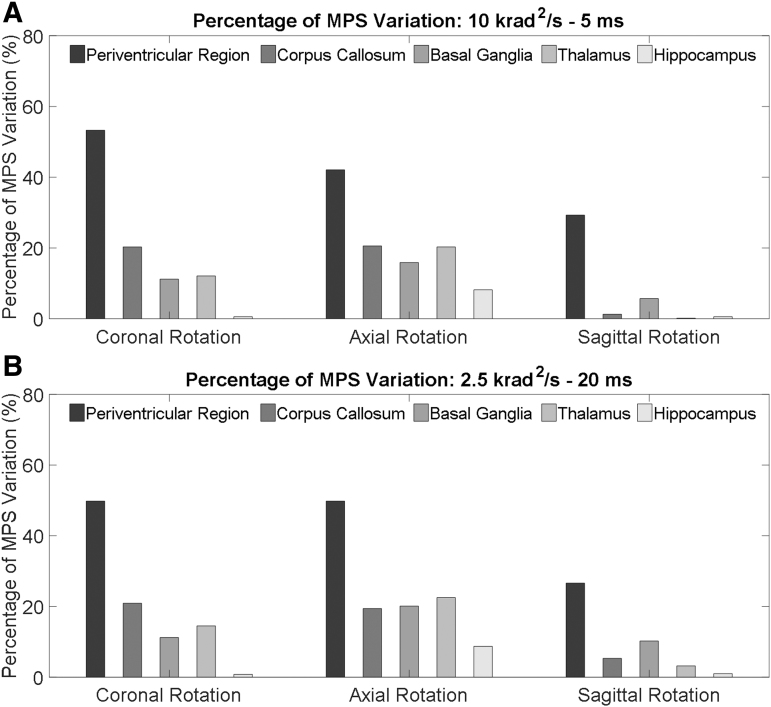
Percentages of the MPS variation in the periventricular region and paraventricular region (i.e., corpus callosum, basal ganglia, thalamus, and hippocampus). The percentages are calculated by using the MPS in the ventricle-model as the baseline. **(A)** Percentage variations of the MPS values in the simulations with the rotational acceleration profiles being the 10 krad/s^2^-5 msec impulse. **(B)** Percentage variations of the MPS values in the simulations with the rotational acceleration profiles being the 2.5krad/s^2^-20 msec impulse. MPS, maximum principal Green-Lagrangian strain.

To further elucidate the ventricle influence on the strain in the periventricular region, brain elements in direct contact with the ventricular CSF (i.e., ventricle wall) were classified into seven regions (Fig. 3A). The MPS in each classified region was compared between the two models. For all the loading conditions, the MPS was exacerbated in the ventricle-model, irrespective of rotation direction and region (Fig. 3B and Fig. 6). The strain-exacerbation effect was further quantified by calculating the percentage of the maximum MPS variation throughout the entire ventricle wall. For the simulations with the rotational acceleration profiles being the 10 krad/s^2^-5 msec impulse, the percentages of MPS variation due to the presence of the ventricles were 53.3%, 42.0%, and 29.3% for coronal, axial, and sagittal rotations, respectively (Fig. 7A). For the simulations with the rotational acceleration profiles being the 2.5 krad/s^2^-20 msec impulse, the percentages of MPS variation were 49.8% for coronal and axial rotations, and 26.6% for sagittal rotation, respectively (Fig. 7B).

**FIG. 8.**
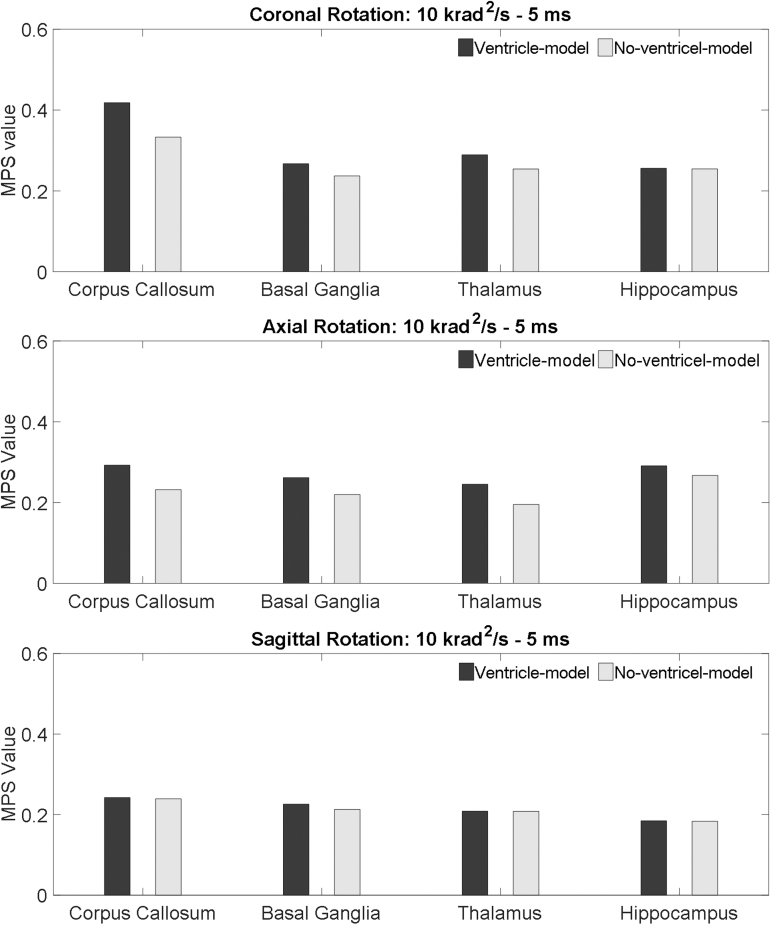
Comparison of the MPS values of the paraventricular region in the ventricle-model and no-ventricle-model for the simulations of coronal rotation, axial rotation, and sagittal rotation, respectively. The rotational acceleration profiles are the 10 krad/s^2^-5 msec impulse for all the simulations. MPS, maximum principal Green-Lagrangian strain.

**FIG. 9.**
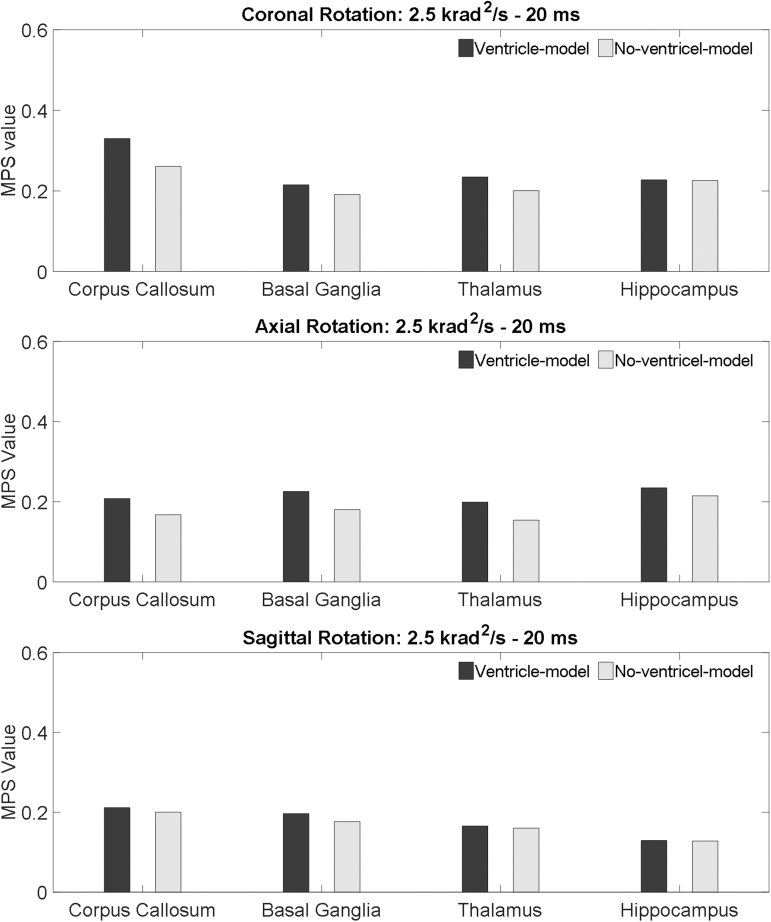
Comparison of the MPS values of the paraventricular region in the ventricle-model and no-ventricle-model for the simulations of coronal rotation, axial rotation, and sagittal rotation, respectively. The rotational acceleration profiles are the 2.5 krad/s^2^-20msec impulse for all the simulations. MPS, maximum principal Green-Lagrangian strain.

MPS in the paraventricular regions, including corpus callosum, basal ganglia, thalamus, and hippocampus, are presented in Figure 8 with the rotational acceleration profiles being the 10 krad/s^2^-5 msec impulse, and Figure 9 with the rotational acceleration profiles being the 2.5 krad/s^2^-20 msec impulse. For all the loading conditions, the MPS in the ventricle-model was larger than that in the no-ventricle-model. Following the same approach of the periventricular region, the strain-exacerbation effect that the ventricles imposed on the paraventricular region was quantified. As characterized by the percentages of the MPS variation, the presence of the ventricles increased strain in the paraventricular region but was less pronounced than that on the periventricular region (Fig. 7).

Results of the Wilcoxon matched pairs signed rank test are summarized in Table 3. For all the ROIs, the *p*-values were less than 0.05, indicating that the MPS in the ventricle-model was significantly larger than that in the no-ventricle-model.

**Table 3.** Mean Percentages in MPS Difference between the Ventricle-Model and No-Ventricle-Model and P-Values

Periventricular region	Mean percentage in MPS difference (%)	*P*	Paraventricular region	Mean percentage in MPS difference (%)	*P*
Region 1: Superior wall of anterior horn	30.8	0.028	Corpus callosum	14.7	0.028
Region 2: Inferior wall of anterior horn	24.9	0.027	Basal ganglia	12.4	0.028
Region 3: Superior wall of ventricle body	39.5	0.028	Thalamus	12.1	0.046
Region 4: Inferior wall of ventricle body	43.2	0.028	Hippocampus	3.3	0.046
Region 5: Superior wall of posterior horn	33.7	0.028			
Region 6: Inferior wall of posterior horn	34.4	0.028			
Region 7: Third ventricle wall	29.2	0.028			

The mean percentages in MPS difference are calculated with the results in the ventricle-model as the baseline. *P*-values for the mean percentages in MPS difference between the ventricle-model and no-ventricle-model are calculated with the Wilcoxon test for matched pairs.

MPS, maximum principal Green-Lagrangian strain.

## Discussion

The present study implemented an FSI approach for the brain–ventricle interface in an anatomically detailed 3D head model. By comparing the strain responses between the two models with and without an inclusion of the cerebral ventricles, it was found that the presence of the ventricles results in strain concentration in the periventricular region. Such a finding provides a plausible biomechanical explanation for the prevalence of periventricular injury in patients with TBI. The presence of the ventricles also increases the strain in the paraventricular region, although less pronounced than that in the periventricular region.

The current prediction of strain concentration encompassing the cerebral ventricles correlates well with the pathological observation of periventricular injury in patients with severe TBI. By classifying the lesion sites of hemispheral sections of 66 brains, Grčević^4^ topologically characterized the predilection sites of periventricular injury, including the lateral walls of lateral ventricular corners, lateral walls of the posterior horns and tips of the frontal horns, and the third ventricle wall. A medicolegal study by Makino^6^ localized the ventricular wall damage by comparing the morphological changes to the ventricle boundary in 50 brain-injured and 50 non-brain-injured cases. The ventricular wall damage was strictly defined as hemorrhagic damage to the subependymal tissue. The damage sites were most frequently observed in the posterior horn, anterior horn, and the attachment of the choroid plexus. Later, another medicolegal study by Kuroda and co-workers^5^ microscopically examined 41 autopsy cases and noted subependymal hemorrhage frequently accompanied by axonal injuries at the anterior horns of the bilateral ventricles in the injured brain. Maxeiner and Schirmer^7^ systematically examined the frequency of intraventricular hemorrhage in 676 formalin-fixed brains and found that ventricular hemorrhages were the predominant results of periventricular lesions. The general topological congruency between current predictions of strain concentrations around the ventricles and the predilection sites of periventricular injury in patients with TBI increases the credence of our computational results.

Periventricular injury, specifically in the form of periventricular axonal injury, has also been observed in animal tests. Of all the experimental efforts, the porcine tests conducted at the University of Pennsylvania are particularly illuminating. By using a custom-built device (HYGE, Inc., Kittanning, PA), rotational accelerations with varying levels of magnitudes were delivered to the porcine head in the coronal plane,^11,15,20–22,61^ axial plane,^22,31^ and sagittal plane.^62,63^ Periventricular axonal injury, identified by axonal retraction balls along margins of the ventricles, was consistently observed. Similar axonal pathology in the periventricular region has also been noted in the rat brain under sagittal rotation^16^ and coronal rotation.^18^ The current prediction of high strain encompassing the ventricles provides a plausible explanation for the prevalence of periventricular axonal injury in the animal tests.

Past FE studies attempted to elucidate periventricular injury mechanisms using different brain–ventricle interaction modeling approaches that, in common, ignored the fluid behavior of the intraventricular CSF. Zhou and colleagues subsequently developed 2D coronal models of the porcine brain^29^ to simulate the experiment by Ross and associates,^20^ and two 3D models of human head with different levels of anatomical differentiation to simulate a frontal impact and a sagittal rotation.^33^ Both studies reported high shear stresses around the ventricles when the ventricles were included. Considering that the ventricles shared interfacial nodes with the brain in both studies, it could be inferred that ventricle perimeters might also endure high shear stresses, which violated the low shear resistance nature of the intraventricular CSF. Alternatively, Miller and co-workers^31^ modeled the ventricles as hollow cavities and allowed the ventricles to collapse. Such a modeling strategy has recently been challenged by Antona-Makoshi^32^ that the cerebrum hemispheres might implode, resulting in error termination or artificial prediction. Further, allowing the ventricles to collapse goes against the incompressible nature of the intraventricular CSF. This study employs an FSI coupling strategy to concatenate the mechanical responses of the ALE-represented ventricle elements with those of the Lagrangian-represented brain elements. This approach not only more realistically reflects the fluid properties of the CSF, but it also circumvents severe fluid mesh distortion by permitting the material flows through the ALE elements.

**FIG. 10.**
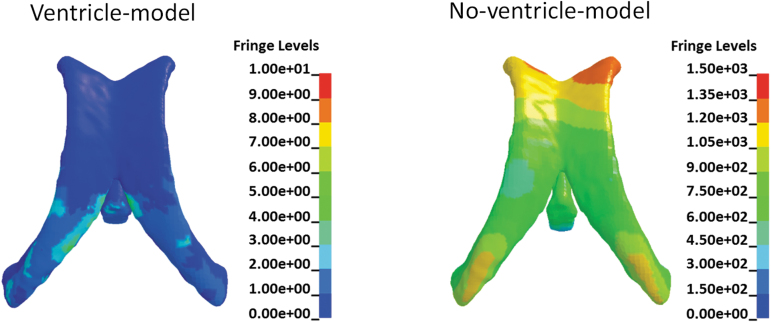
Contour of shear stress in the intraventricular CSF for the ventricle-model and CSF substitute for the no-ventricle-model around the instance maximum value occurs secondary to sagittal rotation with the rotational acceleration profiles being the 10 krad/s^2^-5 msec impulse. Fringe Levels represent shear stress in the unit of Pa. CSF, cerebrospinal fluid; MPS, maximum principal Green-Lagrangian strain.

With implementation of ALE elements for the cerebral ventricles, the deviatoric responses of the intraventricular CSF in the ventricle-model were exclusively determined by its constitutive modeling (Table 3). As exemplified by the results secondary to sagittal rotation with the rotational acceleration profile being the 10 krad/s^2^-5 msec impulse (Fig. 10), the shear stress in the intraventricular substitute in the no-ventricle-model was about 1.5 kPa, providing nonphysical support to the ventricle wall. Comparatively, the shear stress endured by the intraventricular CSF in the ventricle-model was less than 10 Pa, which realistically reflects the low shear resistance properties of the CSF. Thus, the ventricle wall in the ventricle-model was much easier to deform, consequently triggering strain concentration in the periventricular region.

The current study reveals the strain-exacerbation effect that the cerebral ventricles exert on the ventricle wall, both on the superior and inferior partition. Such an effect has been previously reported by Ivarsson and co-workers^27^ based on a 2D physical model, in which the brain strain responses were determined by analyzing the trajectories of the markers embedded within the gel-like brain substitution. However, as critically alluded to by Ivarsson and co-workers,^27^ the marker-based approach for gel strain estimation was not feasible for use in calculating strain locally along the boundary of the ventricles. As a compromise, two marker pairs, deviated from the ventricle boundary for certain distances, were alternatively selected to approximate the anterior-posterior directed tensile strain in the ventricle boundary. Thus, strain in the paraventricular region in fact was reported by Ivarsson and co-workers.^27^. In preference to the physical model, the computational model is amenable to offer spatially detailed strain. Thus, current numerical simulations provide localized information of the periventricular deformation, supporting the hypothesis that the presence of the ventricles results in strain concentration in the periventricular region.

Not limited to the periventricular region, the strain-exacerbation effect associated with the cerebral ventricles is also found in the paraventricular region. This finding does not agree with the conclusions of two previous physical model studies,^26,27^ which reported that the ventricles relieved the strain in the brain regions inferior and superior to the ventricles. Such disagreement may be associated with certain drawbacks in the physical model studies, such as tracking inaccuracy of the marker motion, lack of verification of employing paraffin as a CSF substitute, material variations of the brain substitutes secondary to experimental temperature alteration, as well as approximating the ventricle shape as an idealized ellipse. But it should be clarified that the exact source responsible for this disagreement remains elusive.

**FIG. 11.**
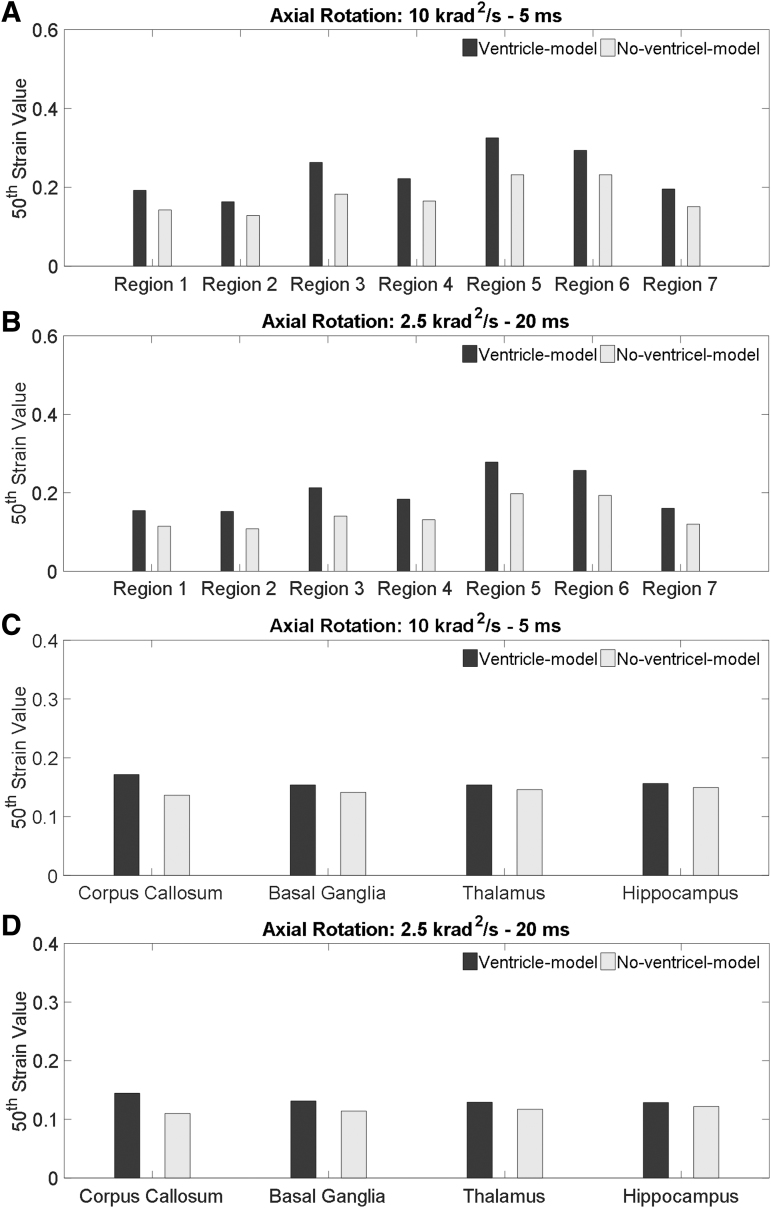
Comparison of the 50th percentile first principal Green-Lagrangian strain predicted by the ventricle-model and no-ventricle-model for the simulations of axial rotations. **(A)** Comparison of the 50th percentile first principal Green-Lagrangian strain in the periventricular region for the simulation with the rotational acceleration profiles being the 10 krad/s^2^-5 msec impulse. **(B)** Comparison of the 50th percentile first principal Green-Lagrangian strain in the periventricular region for the simulation with the rotational acceleration profiles being the 2.5krad/s^2^-20 msec impulse. **(C)** Comparison of the 50th percentile first principal Green-Lagrangian strain in the paraventricular region for the simulation with the rotational acceleration profiles being the 10 krad/s^2^-5 msec impulse. **(D)** Comparison of the 50th percentile first principal Green-Lagrangian strain in the paraventricular region for the simulation with the rotational acceleration profiles being the 2.5krad/s^2^-20 msec impulse.

In this study, the 95th percentile MPS is reported to alleviate the extracted responses in the ROIs from being driven by the response of a single element. As reported by Gabler and colleagues,^64^ compared with the 95th percentile strain, the 50th percentile strain may be more indicative of the average deformation level. Thus, the 50th percentile MPS in the simulations of axial rotations are further checked. As shown in Figure 11, larger strain value is predicted by the ventricle-model, same as the trend revealed when the 95th percentile MPS is used. Such results further reinforce that the presence of the ventricles exacerbates strain in the periventricular and paraventricular regions.

It is worth mentioning that the head model developed in the current study well captures the morphological features of the ventricles based on an octree algorithm,^52^ and is superior to the existing head models with the ventricle shape being either simplified or jagged. Such ananatomically accurate representation of the cerebral ventricles in the FE model enables FSI simulation of the brain–ventricle interaction. Technical details regarding mesh generation based on the octree algorithm are available in previous studies,^65,66^ in which individual-specific pediatric head models were generated.

### Limitations and future work

Although the current study presents some new insights into the periventricular injury mechanism, certain limitations exist that require further investigation and improvement. Due to the limitation of mesh size, the fourth ventricle, as well as the connecting aqueduct, is neglected in the current head models. To the best of our knowledge, only voxel-based models developed by converting each voxel into a single element of the same size maintain these minuscule structures.^44–47^ However, as critically reviewed by Giudice and colleagues,^67^ the voxel-based models exhibit jagged interfaces even with the smoothing operation and are not amenable to FSI implementation. Second, for both models used in the current study, the subarachnoid CSF is modeled as an elastic fluid with the Lagrangian formulation, same as the strategies in other head models,^45–47,68^ in which the morphological heterogeneities of the cerebral cortex in the form of gyri and sulci are presented. Further implementation of the FSI approach for the brain–skull interface in these anatomically detailed head models can contribute to a more accurate representation of the brain–skull interaction. Third, only two rotational impulses with the loading levels approaching the proposed tolerance criteria for DAI in human are used to excite both models in three directions, respectively. Investigation covering a wider range of loading conditions needs to be performed in the future. Following the prevalent loading mode in the animal tests, simulations with inertial loadings were performed in the current study. However, Anderson and colleagues^13^ have also detected periventricular injury in the animal brain in blunt impact with skull deformation. Another possible direction for future work could be extending the current loading regimes to blunt impact scenarios.

## Conclusion

This study investigated the mechanism of periventricular injury with accounting for the fluid behavior of the intraventricular CSF as well as its mechanical interaction with the brain. By comparing the strain responses predicted by two FE models with and without a ventricle inclusion, it was revealed that the presence of the ventricles leads to strain concentration in the periventricular region, which provides a plausible explanation for the prevalence of periventricular injury in patients with TBI. In addition, the strain-exacerbation effect associated with the ventricles was also noted in the paraventricular region, although less pronounced than that in the periventricular region.

## Figures and Tables

**APPENDIX 2 FIG. 1.**
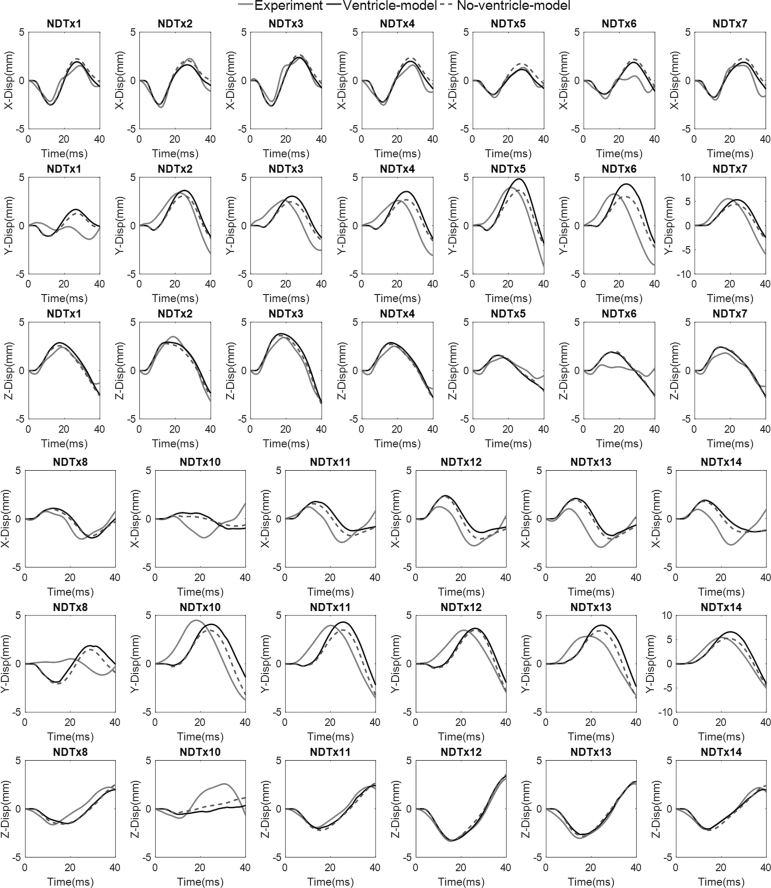
Comparison between experimental motion and simulated brain–skull relative motion in the ventricle-model and no-ventricle-model for the experiment C380-T1.

**APPENDIX 2 FIG. 2.**
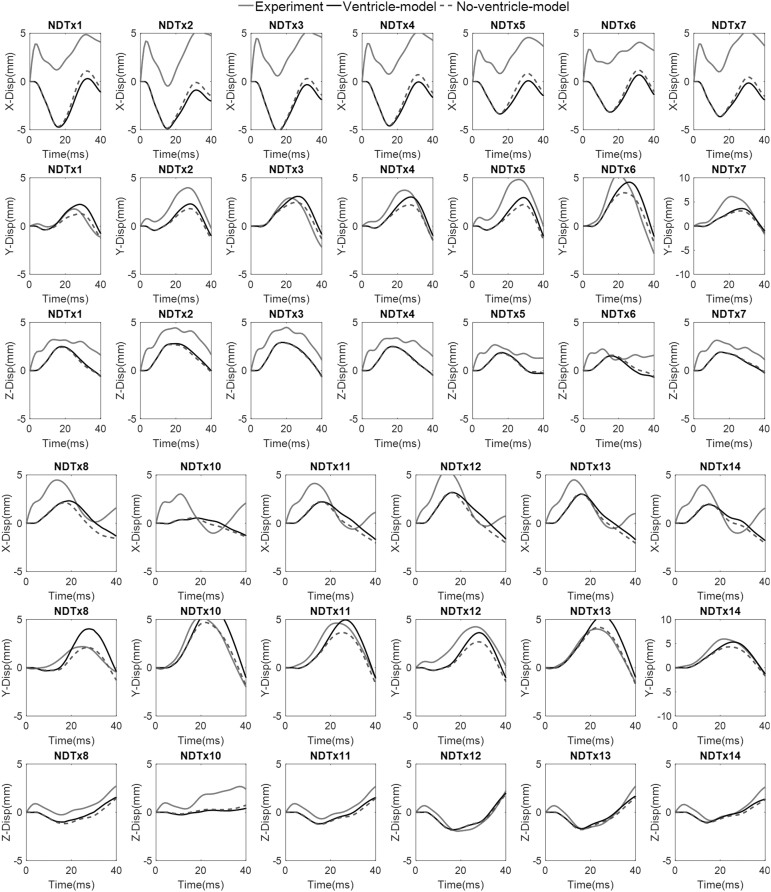
Comparison between experimental motion and simulated brain–skull relative motion in the ventricle-model and no-ventricle-model for the experiment C380-T2.

**APPENDIX 2 FIG. 3.**
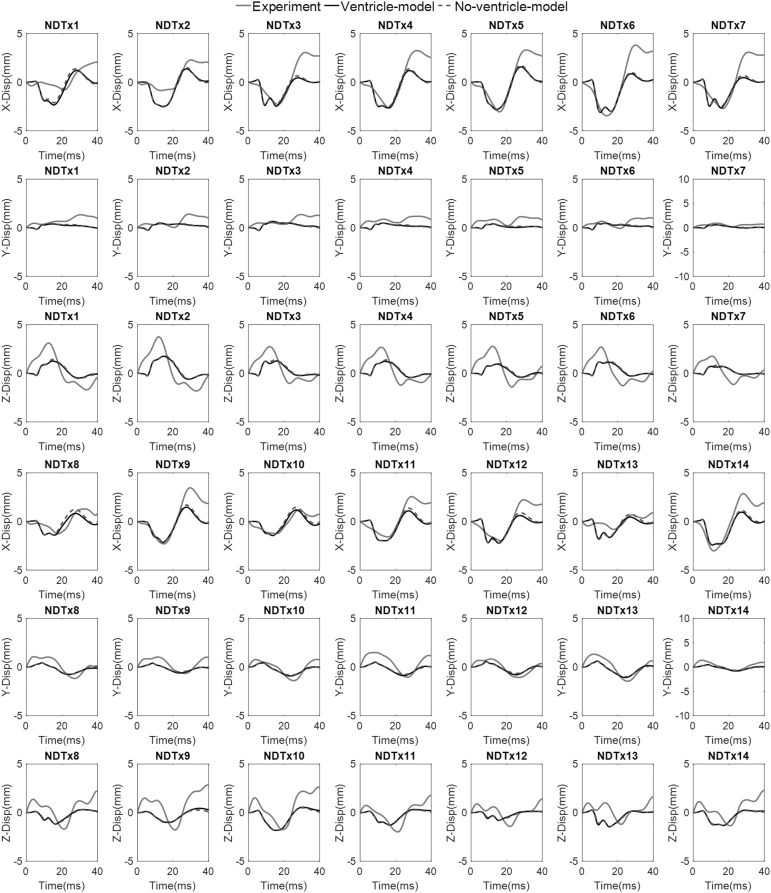
Comparison between experimental motion and simulated brain–skull relative motion in the ventricle-model and no-ventricle-model for the experiment C288-T3.

## Appendix

**Appendix 1.** Element Quality of the FE Model of Human Head

Element type	Jacobian		Warpage		Skew	
Solid element	≥0.5	Minimum	≤30°	Maximum	≤60°	Maximum
	90%	0.12	94%	60°	99%	72°
Shell element	≥0.5	Minimum	≤30°	Maximum	≤45°	Maximum
	99%	0.33	99%	55°	99%	57°

Element type	Minimum angle		Maximum angle		Aspect ratio	
Solid element	≥30°	Minimum	≤150°	Maximum	≤8	Maximum
	99%	13°	99%	168°	99%	12.2
Shell element	≥45°	Minimum	≤135°	Maximum	≤4	Maximum
	99%	25°	99%	168°	99%	5.3

FE, finite element

## Appendix 2

Both models are validated against experimental data of brain–skull relative motion reported by Hardy and co-workers.^56^ Here, three representative experiments are selected, including C288-T3 (sagittal impact), C380-T1 (coronal impact), and C380-T2 (horizontal impact). Details regarding numerical replicating the validating experiments are available in the study by Zhou and colleagues.^69^ Validation results are plotted in Appendix 2 Figures 1–3.
